# Exploring differences in the utilization of the emergency department between migrant and non-migrant populations: a systematic review

**DOI:** 10.1186/s12889-024-18472-3

**Published:** 2024-04-05

**Authors:** Giulia Acquadro-Pacera, Martina Valente, Giulia Facci, Bereket Molla Kiros, Francesco Della Corte, Francesco Barone-Adesi, Luca Ragazzoni, Monica Trentin

**Affiliations:** 1 CRIMEDIM - Center for Research and Training in Disaster Medicine, Humanitarian Aid and Global Health, Universià del Piemonte Orientale, Novara, 28100 Italy; 2 Department for Sustainable Development and Ecological Transition, Università del Piemonte Orientale, Vercelli, 13100 Italy; 3 Department of Translational Medicine, Università del Piemonte Orientale, Novara, 28100 Italy; 4 School of Medicine, Università del Piemonte Orientale, Novara, 28100 Italy

**Keywords:** Migrants, Emergency department, Access to care, Inequalities

## Abstract

**Background:**

Migrants face several barriers when accessing care and tend to rely on emergency services to a greater extent than primary care. Comparing emergency department (ED) utilization by migrants and non-migrants can unveil inequalities affecting the migrant population and pave the way for public health strategies aimed at improving health outcomes. This systematic review aims to investigate differences in ED utilization between migrant and non-migrant populations to ultimately advance research on migrants’ access to care and inform health policies addressing health inequalities.

**Methods:**

A systematic literature search was conducted in March 2023 on the Pubmed, Scopus, and Web of Science databases. The included studies were limited to those relying on data collected from 2012 and written in English or Italian. Data extracted included information on the migrant population and the ED visit, the differences in ED utilization between migrants and non-migrants, and the challenges faced by migrants prior to, during, and after the ED visit. The findings of this systematic review are reported according to the Preferred Reporting Items for Systematic Reviews and Meta-Analyses (PRISMA) 2020 guidelines.

**Results:**

After full-text review, 23 articles met the inclusion criteria. All but one adopted a quantitative methodology. Some studies reported a higher frequency of ED visits among migrants, while others a higher frequency among non-migrants. Migrants tend to leave the hospital against medical advice more frequently than the native population and present at the ED without consulting a general practitioner (GP). They are also less likely to access the ED via ambulance. Admissions for ambulatory care-sensitive conditions, namely health conditions for which adequate, timely, and effective outpatient care can prevent hospitalization, were higher for migrants, while still being significant for the non-migrant population.

**Conclusions:**

The comparison between migrants’ and non-migrants’ utilization of the ED did not suggest a clear pattern. There is no consensus on whether migrants access EDs more or less than non-migrants and on whether migrants are hospitalized at a higher or lower extent. However, migrants tend to access EDs for less urgent conditions, lack a referral from a GP and access the ED as walk-ins more frequently. Migrants are also discharged against medical advice more often compared to non-migrants. Findings of this systematic review suggest that migrants’ access to care is hindered by language barriers, poor insurance coverage, lack of entitlement to a GP, and lack of knowledge of the local healthcare system.

**Supplementary Information:**

The online version contains supplementary material available at 10.1186/s12889-024-18472-3.

## Background

Global migration has been steadily increasing over the past 30 years, with a substantial surge in the number of migrants from 152 million in 1990 to 280 million in 2020 [1]. Migrants, namely people who move away from their place of residence, temporarily or permanently, and for a variety of reasons such as conflicts, work or family issues, [2, 3], may have health needs that are different from those of the general population. In particular, communicable diseases, injuries and trauma, delivery-related complications, as well as mental health issues can result from the harsh conditions experienced throughout the migratory journey [4, 5] and may exert a greater impact on those originating from countries affected by wars, conflicts, or disasters [6]. On the other hand, migrants are often identified as healthier than the host population in light of the “healthy immigrant effect”, which is based on the assumption that people who managed to migrate are more physically fit, younger, healthier, and wealthier [7, 8].

Regardless of their health conditions, migrants, especially those who are undocumented, tend to underutilize healthcare systems compared to the general population [9, 10]. Following Andersen’s expanded behavioral model of health service use [11], the underlying reasons can be clustered in: a) contextual factors, including healthcare organisation and the social, economic and political settings; b) predisposing characteristics, such as demographic attributes; c) enabling factors, which either enable or impede individuals from using healthcare services, such as social and financial resources; d) individuals’ need for healthcare and health needs. Many of these factors coincide with the social determinants of health (SDH), namely non-medical factors that can influence health outcomes and health equity such as income and social protection, unemployment and job insecurity, housing and education [12, 13].

At a systemic level, one of the possible barriers that prevent migrants from using healthcare systems is the lack of migrant-inclusive health policies [14, 15]. Among the many hindering factors it is possible to identify migrants’ financial constraints, limited health literacy, and administrative problems, discriminatory behaviors perpetuated by healthcare professionals, and poor access to health insurance [16]. The fear of being reported to the authorities and deported often prevents irregular migrants from seeking care [17]. Furthermore, language barriers and the lack of professional cultural mediators are also disclosed as reasons for migrants missing medical appointments [18–20]. Migrants may also be unaware of their healthcare rights [21, 22]. Access to care for migrants is further compromised during disasters or public health emergencies, which tend to affect migrants more than the host populations [23–26].

The lack of access to primary health care (PHC) is one of the expressions of migrants’ underuse of the healthcare system as a result of the barriers mentioned above. They may either not have the right to access PHC or be unaware of being entitled to a general practitioner (GP). A short duration of stay in the host country can also prevent registration with a PHC provider. This is particularly problematic as GPs are the entry points to healthcare systems in many countries [27]. A study conducted in Spain in 2016 showed that visits to primary care doctors and nurses were about 50% and 75% less frequent for immigrants than non-migrants [28]. Recent data from England (UK) suggest that the number of GPs and GP funding are lower per capita in more deprived neighborhoods - where migrants presumably live at a higher rate - despite higher health needs in these populations [29]. In the absence of a GP, emergency departments (EDs), accessible around the clock, usually less demanding in terms of bureaucracy and free of charge in many countries, may represent the best option for migrants seeking medical advice [30, 31]. Migrants who have access to PHC may encounter difficulties in visiting a doctor during normal working hours as they are typically employed in informal and inflexible jobs. Due to the poor use of primary and preventive care services, migrants are expected to overuse the ED, especially for lower acuity and non-urgent conditions [31–33]. Therefore, EDs constitute a unique healthcare setting, as they are situated at the interface of outpatient and inpatient care [34]. Studying their utilization is relevant because it reflects the need for urgent care and is an indicator of the accessibility and quality of outpatient and hospital-based care [35]. In other words, investigating migrants’ use of the ED can provide a glimpse into their relationship with the healthcare system of the host country and into the obstacles they may face.

Studies dealing with the utilization of the ED by migrant populations often lack comparisons with host populations [30, 36–44]. Yet, such a comparative method would capture the relevant inequities existing between migrants and the general population in terms of health-seeking behavior, barriers to accessing ambulatory care, relationship with healthcare professionals, clinical outcomes and quality of care received. As for reviews of the literature dealing with migrants’ utilization of the ED, these are either country-specific [45] or limited to the European context [27, 46, 47]. Conversely, the review of Mahmoud et al. [48] considers studies conducted worldwide, but was published in 2012 and it is therefore outdated, as many new articles have been published since then.

The aim of this systematic literature review is to gather and summarize published literature that compares ED utilization between migrant and non-migrant populations to identify differences in access to care and utilization of the ED. This systematic review will provide decision-makers with relevant information that can support the design of healthcare policies, practices, and interventions addressing migrants’ inequities. This is even more pressing considering that over the next 30 years approximately 143 million people will be displaced due to the consequences of climate change [49], while others are expected to migrate for other causes, such as non-climate-related disasters, wars, conflicts, environmental degradation, and poverty.

## Methods

The Preferred Reporting Items for Systematic Reviews and Meta-Analyses (PRISMA) 2020 guidelines were followed for reporting the findings of this systematic review [50].

### Search strategy

A systematic literature search was conducted on March 20th 2023, on the PubMed, Scopus, and Web of Science databases. The search strings (Supplementary material 1) combined two different sets of terms, namely migrant-related and ED-related ones. No restrictions or filters were applied for the search. After the removal of duplicates, titles and abstracts of the remaining articles were manually screened by three investigators (GAP, GF, BMK) and those not meeting the inclusion criteria were excluded. All the full-text articles eligible for inclusion were reviewed independently by three investigators (GAP, GF, BMK) and discrepancies were resolved after discussion with the whole group. The references of the selected articles were also screened to identify any other relevant studies to be included.

### Eligibility criteria

The study selection process relied on the following inclusion criteria: *a*) the study included a comparison between migrants and non-migrants regarding the utilization of the ED; *b*) the study relied on data collected over the period 2012 - March 2023; *c*) the study was original research, adopting either a quantitative, qualitative or a mixed-methods methodology; *d*) the article is either in English or Italian. Exclusion criteria were: *a*) the study is not about migrants’ utilization of the ED; *b*) the study does not include any comparison between migrants and non-migrants regarding the utilization of the ED; *c*) the study is about migrants’ utilization of pre-hospital emergency medical services; *d*) the study does not distinguish between data on the use of the ED and data related to other levels of care; *e*) the study is a clinical case study; *f*) the study is a review or a commentary; *g*) the study is not in English or Italian.

### Data extraction, analysis, and reporting

A data extraction sheet was developed to extract relevant information from the included studies (Supplementary material 2). Data extraction was performed by two investigators (GAP, GF). Extracted data included, among others, information on the article’s main characteristics and the study design, information about the migrant population and the ED visit, the differences in the ED utilization between migrants and non-migrants, and information about the challenges faced by migrants prior to, during, and following the ED visit.

After demographic information, the differences in ED utilization between migrant and non-migrant populations are reported following four main themes: *i*) access to the ED; *ii*) adequacy of utilization of the ED; *iii*) reasons for accessing the ED; *iv*) hospitalization and discharge. Different types of barriers to access to care and health systems’ characteristics are reported in Supplementary material 3.

For operational purposes, the term “migrant” is used in its broadest sense to refer to people who move away from their place of usual residence across an international border, temporarily or permanently, for a variety of reasons such as war, family issues or work [2]. To account for the peculiarities of different migratory experiences, the definition of migrant as reported by the authors of the original studies (e.g., asylum-seeker, refugee, etc.) has been specified when possible.

## Results

**Fig. 1:**
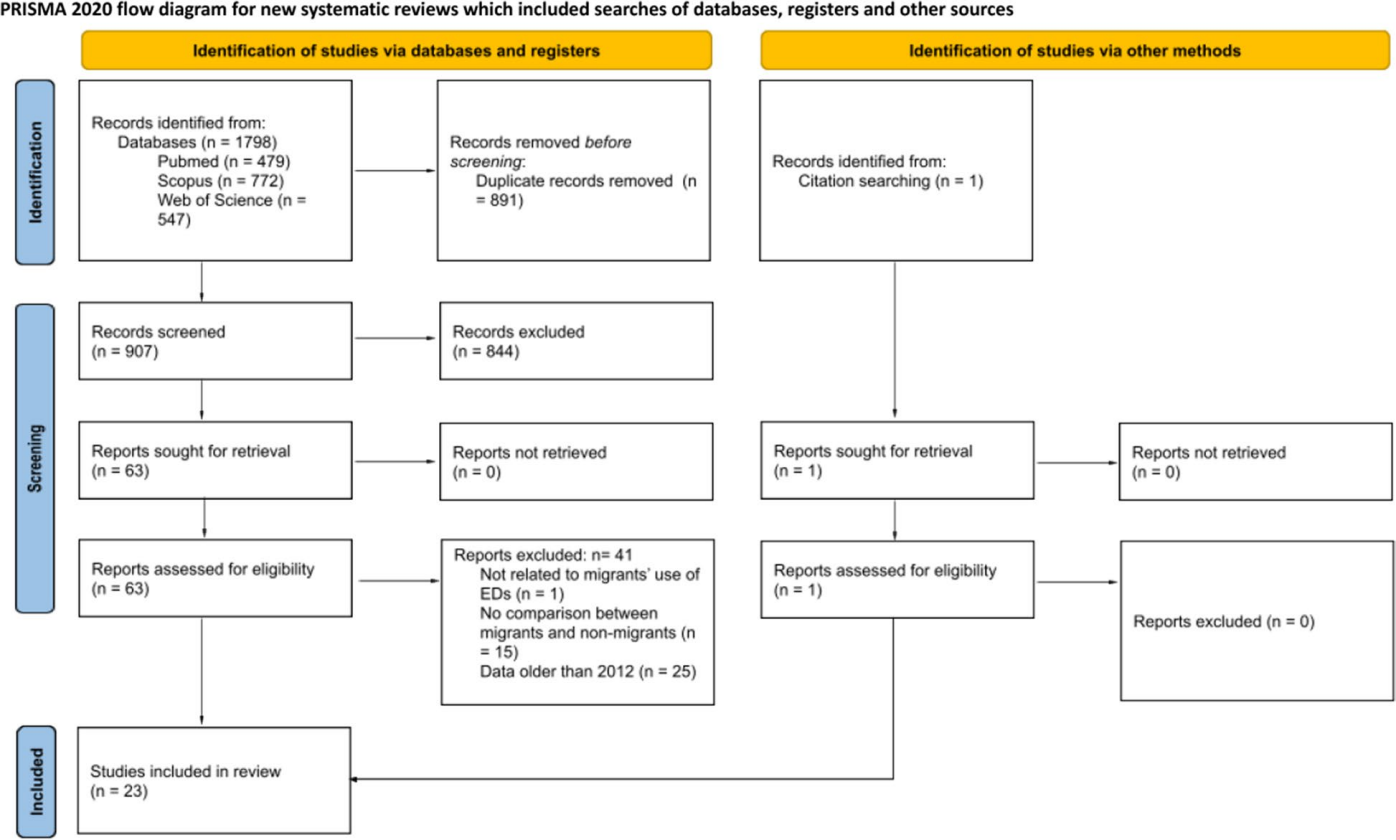
Study selection process

The search returned a total of 1,798 articles. After removing duplicates, 907 articles were eligible for title and abstract review. Among these, 844 were excluded because they did not meet our inclusion criteria. One article was identified through manual search. In total, 64 articles met the criteria for full-text review. After full-text review, 23 articles were included. Detailed information regarding the selection of articles can be found in the PRISMA diagram (Fig. 1), while a comprehensive overview of the main characteristics of the studies is presented in Supplementary material 4.

### Characteristics of the studies

Among the included studies, 22 adopted a quantitative approach, whereas only one [51] used a qualitative methodology. Among the quantitative studies, 16 were cross-sectional, 4 were observational, and 2 were cohort studies. The qualitative study adopted a grounded theory approach. Sources of data primarily included hospital medical records, population surveys, and interviews (Table 1). The studies included in this review were conducted in 12 countries: United States (US) (*n* = 5), Switzerland (*n* = 4), Germany (*n* = 3), Italy (*n* = 2), Spain (*n* = 2), Australia (*n* = 1), Canada (*n* = 1), China (*n* = 1), France (*n* = 1), Lebanon (*n* = 1), Singapore (*n* = 1), and Türkiye (*n* = 1).

**Table 1:** Characteristics of the included studies

Characteristics	Number of studies	References
**Host Countries**		
US	5	[51–55]
Switzerland	4	[56–59]
Germany	3	[60–62]
Italy	2	[63, 64]
Spain	2	[65, 66]
Australia	1	[67]
Canada	1	[68]
China	1	[69]
France	1	[70]
Lebanon	1	[71]
Singapore	1	[72]
Türkiye	1	[73]
**Population characteristics**		
Migrant definition		
Immigrant	7	[59, 63–68]
Migrant	5	[52, 61, 62, 69, 70]
Asylum seeker	4	[56–58, 60]
Undocumented	4	[51, 53–55]
Refugee	2	[71, 73]
Foreign worker	1	[72]
Place of birth		
Only region reported	10	[51–55, 58, 61, 66, 70, 73]
Not reported	7	[60, 62–65, 68, 69]
Country reported	6	[56, 57, 59, 67, 71, 72]
**Study design**		
Quantitative	22	[52–73]
Qualitative	1	[51]
**Data collection**		
Retrospective	18	[52–57, 59–61, 63–66, 68, 70–73]
Prospective	5	[51, 58, 62, 67, 69]
**Source of data**		
Hospital records	12	[52–54, 56, 57, 59, 60, 63, 64, 71–73]
Surveys	7	[55, 58, 65–69]
Interviews	3	[51, 61, 62]
Multiple sources	1	[70]

### Demographic characteristics of migrants

**Fig. 2:**
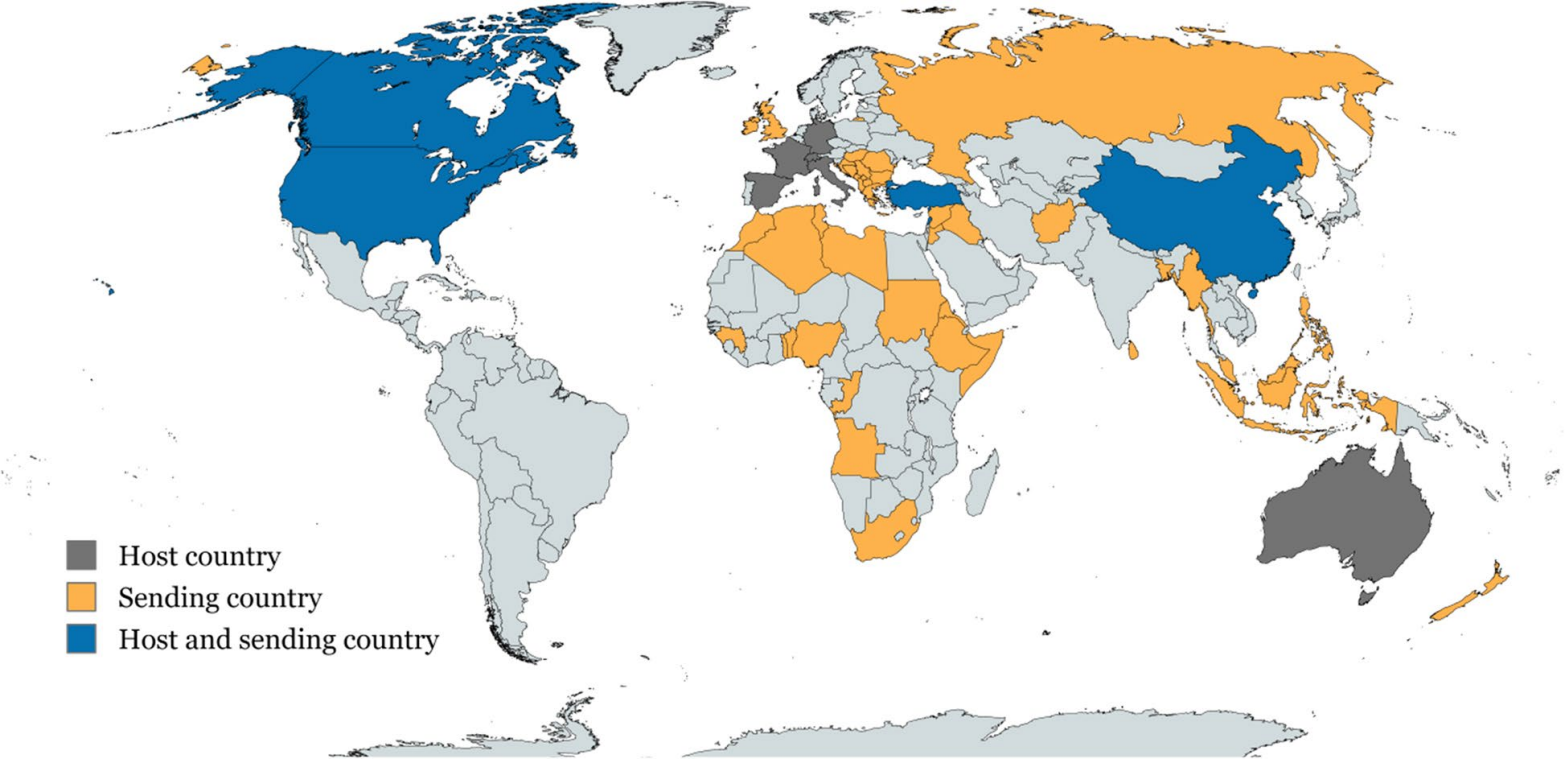
Map illustrating host and home countries according to the information reported in the included studies

The studies included in this review refer to their target population as “immigrants” [59, 63–66, 68], “migrants” [52, 61, 62, 69, 70], “asylum seekers” [56–58, 60], “undocumented” [51, 53–55], “refugees” [71, 73], and “foreign workers” (FWs) [72]. For studies employing interviews or surveys, migratory status was primarily based on self-reported information. Only four articles reported information about migrants’ length of stay in the host country, whereas one study distinguished between first and second-generation immigrants (Supplementary material 5). Migrants’ country of origin was reported in six articles, whereas ten reported the broader area or region. A map illustrating migrants’ home and host countries is reported in Fig. 2.

Information on migrants’ age was not always reported. Pediatric patients were the focus of three studies [56, 57, 70], while another study included both pediatric patients and their mothers [52]. One study specifically focused on migrants aged 60 and above [69]. For further information about participants’ age, see Supplementary material 5.

### Differences in ED utilization between migrants and non-migrants

#### Access to the ED

The results show varying patterns concerning the frequency and likelihood of ED visits by migrants compared to non-migrants (Table 2).

**Table 2:** Records concerning migrants’ access to the ED

Access to the ED					
Record	Country and classification ^a^	Migrant sub-population of interest	Measures of frequency	Measures of association	Direction of association with migrant status
Abdulla et al. (2020) [52]	US. (HIC)	Mothers of preterm infants	Immigrants vs. Italian natives: males (371 vs 309), females (365 vs 299).	Crude OR: 1.7 (1.12 - 2.59)	$\uparrow$
				Adjusted OR: 2.42 (0.95 - 6.19)	
Di Napoli et al. (2022) [63]	Italy (HIC)	No sub-population considered	Immigrants vs. Italian natives (age-standardized rates per 1,000 residents): males (371.8 vs. 309.2), females (365.3 vs. 299.4).	n/a	$\uparrow$
Rodriguez-Alvarez et al. (2019) [66]	Spain (HIC)	No sub-population considered	Immigrants vs. Spanish natives: 19.3% vs. 9.9%.	Male immigrants - Crude PR: 1.70 (1.33-2.18)	$\uparrow$
				Adjusted PR: 1.50 (1.01-2.25)	
				Female immigrants - Crude PR: 2.08 (1.72-2.51)	
				Adjusted PR: 1.97 (1.43-2.69)	
Ornelas et al. (2021) [51]	US (HIC)	No sub-population considered	n/a	n/a	$\downarrow$
Brandenberger et al. (2021) [57]	Switzerland (HIC)	Children	Asylum-seeking children vs. non-asylum-seeking children: 19% vs. 32%.	n/a	$\downarrow$
Henares-Montiel et al. (2018) [65]	Spain (HIC)	No sub-population considered	Male immigrants vs. Spanish natives: 24.5% vs. 24.7%.	Male immigrants, adjusted OR: 0.95 (0.79-1.12)	$=$
			Female immigrants vs. Spanish natives: 29.8% vs. 29.3%.	Female immigrants, adjusted OR: 0.97 (0.84-1.13)	

^a^According to the World Bank

* HIC* High Income Country, *OR* Odds Ratio, *PR* Prevalence Ratio

A total of three studies showed either a higher utilization of the ED from migrants or an increased probability of migrants accessing the ED than the host population. The study of Abdulla et al. [52] considered a group of immigrant mothers and their preterm infants seeking care at the ED in the US and found that infants of immigrant mothers were more likely to visit the ED in the first 30 and 90 days after being discharged (odds ratio (OR): 1.7; 95%CI: 1.12-2.59), as compared to those of non-immigrant mothers. However, when considering mothers with Medicaid coverage - namely an insurance program for people with limited income and resources - the immigrant status in relation to high ED utilization lost significance, suggesting that the higher risk of ED visits for preterm infants may be due to stressors like poverty. A retrospective analysis comparing ED utilization between immigrants and Italian citizens [63] found a higher frequency of visits to the ED among immigrants than Italians. The authors ascribe this finding to immigrants’ poor familiarity with the host country’s healthcare system, compounded by complex bureaucracy and language barriers. Similarly, Rodriguez-Alvarez et al. [66] found that, compared to their native counterparts, immigrants used the ED to a greater extent (19.3% vs. 9.9%; *p*-value < 0.001). The authors attribute this trend to factors such as easy accessibility, the services being free of charge, and their 24-hour availability.

Conversely, two studies found a lower utilization of the ED from migrants than the host population. Brandenberger et al. [57] found that the proportion of asylum-seeking pediatric patients visiting the ED in Switzerland was lower than that of non-asylum-seeking patients (19% vs. 32%; *p*-value < 0.001); in this regard, it must be clarified that, regarding ED access, nationality was unspecified for the non-asylum-seeking group, thus some non-asylum-seeking migrants (i.e., refugees and undocumented children) may have been grouped together with Swiss nationals. In another qualitative study conducted in the US, migrants’ low utilization of the ED has been attributed to their fear of discrimination, denial of services, and law enforcement in the hospital in the years following the 2016 US presidential elections [51].

Last, Henares-Montiel et al. [65] compared immigrants and the host population in Spain, finding very similar percentages of ED utilization across the two groups (24.5% vs. 24.7%; *p*-value > 0.05); nevertheless, the relationship was not statistically significant.

When it comes to the type of hospital, Al-Hajj et al. [71] examined injured patients presenting to the ED and found that almost 90% of Lebanese patients sought care at private hospitals, as compared to only 52% of refugees (*p*-value < 0.001). According to the authors, a reason for this difference is that refugees are frequently unable to pay for medical care and therefore tend to rely to a greater extent on public hospitals or other facilities sponsored by local non-governmental organizations or the United Nations High Commissioner for Refugees (UNHCR).

#### Adequacy of utilization of the ED

In total, three factors have been considered as indicative of the adequacy of ED utilization: *a*) urgency/appropriateness, *b*) admissions for ambulatory care-sensitive conditions (ACSC), *c*) self-referral and walk-in access (Table 3). The results show varying patterns; however, they suggest a lower adequacy of ED utilization by migrants compared to non-migrants.

**Table 3:** Records concerning the adequacy of utilization of the ED

Adequacy of utilization of the Emergency Department (ED)					
Record	Country and classification^a^	Migrant sub-population of interest	Measures of frequency	Measures of association	Direction of association with migrant status
**Urgency/Appropriateness**					
Schwachenwalde et al. (2020) [62]	Germany (HIC)	Women	n/a	Low-acculturated migrants vs. German natives for system-defined non-urgent gynecologic ED use: Adjusted OR 1.58 (1.02-2.44).	$\downarrow$
Chan et al. (2021) [72]	Singapore (HIC)	No sub-population considered	Foreign workers triaged as low-acuity vs. general ED population: 66.9% vs 45.9%.	n/a	$\downarrow$
Klukowska-Roetzler et al. (2018) [59]	Switzerland (HIC)	Southeast Europe natives	Mean triage level (scale of 1-5, most-less urgent) of southeast immigrants vs. Swiss citizens: 2.84 (2.82-2.85) vs. 2.61 (2.60-2.61).	n/a	$\downarrow$
Sauzet et al. (2021) [61]	Germany (HIC)	No sub-population considered	n/a	1st generation migrants vs. German natives: Adjusted OR 0.72 (0.57, 0.91).	$\downarrow$
				2nd generation migrants vs. German natives: Adjusted OR 0.76 (0.53, 1.09).	
**Admissions for Ambulatory Care-Sensitive Conditions (ACSC)**					
Brandenberger et al. (2020) [56]	Switzerland (HIC)	Children	Ambulatory care sensitive conditions (ACSC) admissions in asylum-seeking children vs. non-asylum-seeking children: 12.1% (0.07-0.18) vs. 10.9% (0.1-0.11).	n/a	=
Lichtl et al. (2017) [60]	Germany (HIC)	Children	ED visits for ACSC in asylum-seeking children vs. non-asylum-seeking children: 29.5% vs. 7.9%.	ED visits for ACSC in asylum-seeking children vs. non-asylum seeking children, crude OR: 4.89 (4.1-5.85) adjusted OR: 4.93 (4.11; 5.91).	$\uparrow$
**Self-referral and walk-in access**					
Klukowska-Roetzler et al. (2018) [59]	Switzerland (HIC)	Southeast Europe natives	Southeast migrants self-referring vs. Swiss nationals: 59.9% vs. 41.2%.	n/a	$\uparrow$
Mahmoud et al. (2015) [67]	Australia (HIC)	Non-English speaking non-native patients	n/a	Non-English speaking non-native patients contacting a general practitioner before accessing vs. Australian natives: OR: 0.6 (0.4-0.8).	$\uparrow$
Chan et al. (2021) [72]	Singapore (HIC)	No sub-population considered	Foreign workers arriving by ambulance vs. general ED population: 6.1% vs. 13.3%.	n/a	$\uparrow$
Klingberg et al. (2020) [58]	Switzerland (HIC)	No sub-population considered	Asylum seekers without a general practitioner contact prior to access vs. Swiss nationals: 63.2% vs. 67.6%.	n/a	$\downarrow$

^a^According to the World Bank

* HIC* High Income Country, *OR* Odds Ratio

##### Urgency/Appropriateness

All the studies dealing with urgency and/or appropriateness of the ED visit report migrants’ accesses as being less urgent compared to non-migrants (Table 3). Klukowska-Roetzler et al. [59] found a significant association between triage level and immigration from South Eastern Europe, with migrants being assigned lower triage codes, meaning that they were categorized as having less urgent medical needs than native Swiss patients. The same authors also noted how there were more migrants from Southeast Europe (18.9%) utilizing fast-track services, designed for less serious illnesses and injuries, compared to Swiss nationals (9.9%). Schwachenwalde et al. [62] identified low-acculturated migrant women (acculturation was assessed by the Frankfurt Acculturation Scale [7]) seeking gynecology emergency care in Germany as more likely to visit the ED for non-urgent care as compared to non-migrant women (OR: 1.58; 95%CI: 1.02-2.44). When analyzing the impact of acculturation on overall non-urgent healthcare utilization among migrants, the authors found no significant difference as compared to non-migrants. However, low acculturation emerged as a significant positive predictor of system-defined non-urgent visits, meaning visits categorized as non-urgent based on health system criteria, such as no ambulance transport, absence of a referral by a physician, and not resulting in hospital admission. On the other hand, low acculturation represented a negative predictor of patient-defined non-urgent visits, categorized based on subjective criteria such as low level of pain or symptom severity, and low estimation of urgency by the patient. Such findings underline the difficulty of defining urgency and the authors speculate that the inappropriate use of the ED by migrants can be attributed both to the patients’ distorted perception and to deficiencies in the provision of care (e.g., bias and language barriers). Sauzet et al. [61] investigated the adequacy of the use of ED services in Germany, considering whether patients were sent by medical professionals, reported severe pain, or had a medical urgency. The authors found that first-generation migrants were significantly less likely to appropriately use the ED compared to non-migrants. Similarly, Chan et al. [72] found that FWs living in Singapore were significantly more often triaged as low-acuity patients compared to the general ED population.

Rodriguez et al. [55] report a higher fear of accessing the ED among undocumented Latino immigrants (UDLI) compared to non-Latino legal residents/citizens (NLRC) (UDLI 24%, 95% CI 20-28% vs. NLRC 4%, 95% CI 2-6%) after the anti-immigrant statements made during the 2016 US presidential campaign. The authors found that this fear ultimately led migrants to delay care, which could suggest migrants presented with more urgent conditions, contrary to what the other studies have reported.

##### Admissions for ACSC

Admissions for ACSC, namely medical conditions for which hospitalization is not needed when primary care is timely and effective, occurred more for migrant populations, as compared to host populations (Table 3). Brandenberger et al. [56] found that, in Switzerland, 10.74% of asylum-seeking pediatric patients’ admissions were for ACSC and happened via the ED, while the percentage dropped to 9.45% for the host population. Similarly, Lichtl et al. [60] found that asylum-seeking pediatric patients were 4.89 times (95%CI: 4.10-5.85) more likely to use emergency outpatient services for ACSC than the general population in Heidelberg (Germany), with children up to three years old being the most likely to use the ED for ACSC (OR: 1.19; 95%CI: 1.0-1.42). The authors mention as a possible explanation for this finding how asylum seekers might have insufficient knowledge and information on the host country’s health system, which may lead to utilizing emergency outpatient services even for conditions that could be treated at a primary care level.

##### Self-referral and walk-in access

When it comes to the modality of referral, the included studies report a trend toward increased self-referrals, and walk-in accesses, by migrants compared to non-migrants (Table 3). Klukowska-Roetzler et al. [59] found a higher percentage of Southeast European migrants visiting the ED upon self-referral compared to Swiss patients (59.9% vs. 41.2%), which were instead referred by ambulance to a greater extent (16.2% vs. 7.7%). Similarly, Mahmoud et al. [67] compared ED utilization across three groups, namely non-English speaking non-native patients (NESB), English-speaking non-native patients (ESB-NBA), and English-speaking native Australian patients (ESB-BA) and found that NESB patients were less likely to contact a GP before seeking care at the ED compared to ESB-BA patients (OR: 0.6; 95%CI: 0.4-0.8). These findings are in agreement with those from Chan et al. [72] who found a significantly lower percentage of FWs arriving by ambulance compared to the native population in Singapore (6.1% vs. 13.3%; *p*-value < 0.001). As brought about by Di Napoli et al. [63] in a study conducted in Italy, the limited working hours of GPs may represent a barrier to accessing primary care services, especially for those people having precarious working conditions. Yet, the results of Klingberg et al. [58] go in the opposite direction, as they found a smaller percentage of asylum seekers visiting the ED without prior consultation with a GP than Swiss patients (63.2% vs. 67.6%).

#### Reasons for accessing the ED

Al-Hajj et al. [71] found that refugees experienced a higher proportion of occupational injuries compared to Lebanese nationals (12.4% vs. 4.9%, *p*-value < 0.001) and explain this difference by noting how the refugee’s male workforce may be exposed to hazardous workplace conditions in industrial or construction sites, which may increase their likelihood of being injured. The regression analysis also shows that being a refugee increases the odds of sustaining cuts/bites/open wounds (OR: 1.30; 95%CI: 1.07-1.58), concussion (OR: 1.72; 95%CI: 1.15-2.57), gunshot or stab injuries (OR: 3.392, 95%CI=2.605-4.416), and organ system injury (OR: 1.77; 95%CI: 1.16-2.7), as well as lower odds for presenting with a bruise (OR: 0.74, 95%CI: 0.61-0.90).

Ro et al. [54] compared the ED visits between undocumented migrants and individuals covered by MediCal, an insurance scheme that covers individuals with low income, both natives and authorized foreign-born individuals. The authors identified higher odds of having a COVID-19-related ED visit among young undocumented patients than young MediCal patients (OR: 1.37; 95%CI: 1.24-1.52). Similarly, Huynh et al. [53] further expanded the analysis by comparing ED visits for COVID-19 between undocumented migrants and MediCal patients over time, finding higher percentages of COVID-19-related visits in the former (5.9% vs. 3.7%). The authors reject the hypothesis that undocumented patients were over-reliant on EDs compared to MediCal patients: a sensitivity check highlighted how undocumented migrants were less likely to go to the ED for heart failure than MediCal patients (OR: 0.66; 95%CI: 0.55-0.79) in the same period. Thus, it appears that the differences in ED utilization for COVID-19-related needs have to be ascribed to higher rates of COVID-19 infections among undocumented patients. The choice of MediCal patients as a comparison group implies that authorized foreign-born individuals are analyzed together with US citizens, posing potential issues in the interpretation of results. However, the Public Policy Institute of California reports that applicants may face waiting periods of several years to become legal permanent residents [74]; thus, we assume that a longer stay in the country would be a proxy for a higher level of knowledge of the functioning of the healthcare system. This likely leads to a health-seeking behavior more similar to that of US citizens compared to undocumented immigrants.

#### Hospitalization and discharge

The results show varying patterns concerning ED contacts resulting in hospitalization, as well as discharge, for migrants compared to non-migrants (Table 4).

Klukowska-Roetzler et al. [59] showed that immigrants from Southeast Europe were hospitalized to a lesser extent than native Swiss patients (21.0% vs. 34.5%), yet those triaged with more urgency had a higher hospitalization rate. Al-Hajj et al. [71] found lower hospitalization rates for refugees as compared to local Lebanese patients (7.1% vs. 10.3%; *p*-value = 0.018) and, along the same lines, Zunino et al. [70] found lower hospitalization rates for migrant children in France, as compared to children from the local population (9% vs. 14.6%). As for the latter, it is important to acknowledge a significant selection bias, as migrant children with more serious health conditions were not counted in emergency visits; yet, these findings seem to agree with migrants receiving lower triage codes.

Conversely, Abdulla et al. [52] found higher hospitalization rates for infants of immigrant mothers compared to natives in the US (13% vs. 8%; *p*-value = 0.06), proposing illness severity, challenges with communication or discharge planning as possible reasons. Brandenberger et al. [56] found that the proportion of ED contacts leading to admission was higher in asylum seekers compared to non-asylum seekers (25% vs. 10%).

Huynh et al. [53] compared ED visits for COVID-19 between undocumented migrants and MediCal patients, finding undocumented patients to be as likely to have a visit resulting in admission as MediCal patients (OR: 1.05; 95%CI: 0.80-1.38).

Al-Hajj et al. [71] found a higher percentage of refugees leaving the hospital Against Medical Advice (AMA) compared to locals (5.6% vs. 2.8%, *p*-value < 0.001). The authors explained the finding by mentioning refugees’ limited access to health care and limited resources, which could result in them being unable to sustain the costs associated with hospital admission. Similarly, Chan et al. [72] found that AMA discharges for FWs visiting the ED in Singapore were more numerous than for the general population (11.3% vs. 4.3%; *p*-value < 0.001), with the majority of AMA discharges being for non-trauma-related conditions. These findings could further justify lower hospitalization rates among migrant populations.

As for the length of stay in the ED, Klingberg et al. [58] examined emergency care utilization of asylum seekers in Switzerland and found a shorter median length of stay for asylum seekers as compared to the host population (3.09h vs. 3.22h; *p*-value = 0.141). On the opposite, Gulacti et al. [73] assessed ED utilization by Syrian refugees in Türkiye and found that the median length of stay in the ED was significantly longer for refugees than for the host population (8.54h vs. 5.95; *p*-value < 0.001). Similarly, Zunino et al. [70] found that the average length of stay for migrants was 3.9h, slightly longer than visits of other patients (*p*-value < 0.025). Language and communication barriers, with limited use of interpreters, could significantly influence the length of stay in the ED [58, 70].

**Table 4:** Records concerning hospitalization from the ED and discharge

Emergency Department (ED) contact resulting in hospitalization					
Record	Country and classification^a^	Migrant sub-population of interest	Measures of frequency	Measures of association	Direction of association with migrant status
**Hospitalization**					
Klukowska-Roetzler et al. (2018) [59]	Switzerland (HIC)	Southeast Europe natives	Southeast immigrants vs. Swiss natives: 21.0% vs. 34.5%.	n/a	$\downarrow$
Al-Hajj et al. (2021) [71]	Lebanon (MIC)	Individuals presenting with an injury	Refugees vs. Lebanese natives: 7.1% vs. 10.3%.	n/a	$\downarrow$
Zunino et al. (2021) [70]	France (HIC)	Children	Migrant children vs. children of the general population: 9% vs. 14.6%.	n/a	$\downarrow$
Brandenberger et al. (2020) [56]	Switzerland (HIC)	Children	Asylum seeking children vs. non-asylum seeking children: 25% vs. 10%.	n/a	$\uparrow$
Abdulla et al. (2020) [52]	US (HIC)	Mothers of preterm infants	Infants of immigrant mothers vs. infants of US natives: 13% vs. 8%.	n/a	$\uparrow$
Huynh et al. (2023) [53]	US (HIC)	No sub-population considered	n/a	Undocumented patients vs. MediCal patients OR: 1.05 (0.80-1.38).	=
**Leaving the hospital against medical advice**					
Chan et al. (2021)[72]	Singapore (HIC)	No sub-population considered	Foreign workers vs. general population: 11.3% vs. 4.3%.	n/a	$\uparrow$
Al-Hajj et al. (2021) [71]	Lebanon (MIC)	Individuals presenting with an injury	Refugees vs. Lebanese natives (5.6% vs. 2.8%).	n/a	$\uparrow$

^a^According to the World Bank

* HIC* High Income Country, *MIC* Middle Income Country, *OR* Odds Ratio

## Discussion

This systematic review gathered and summarized published literature highlighting differences in ED utilization between migrant and non-migrant populations. Our findings did not suggest a single pattern regarding migrants’ access to and use of EDs. Some studies [52, 63, 66] reported a higher frequency and/or likelihood of ED visits among migrants, while others [57, 69] reported a higher frequency and/or likelihood of ED visits among non-migrants. Some authors agree on the fact that migrants tend to visit the ED for less urgent conditions compared to the host populations [59, 62, 63, 72]; however, there is disagreement about whether migrants are more [52, 57], or less [59, 70, 71] hospitalized than non-migrants. Migrants are consistently reported as being more prone to leaving the hospital AMA than the host population [71, 72]; more consistency was found regarding the mode of access: compared to non-migrants, migrants seek care at the ED without consulting a GP first more often [58, 67] and access the ED via ambulance less often [59, 72].

Several considerations can be made in regard to the lower reliance on ambulances. The first one is associated with contextual factors, as in countries where the cost of ambulance services is covered only for urgent conditions, like Singapore or Switzerland, migrants of low socioeconomic status (SES) may be unwilling to take the risk of being charged. Second, migrants may be impeded from using this service because i) calling an ambulance implies knowing the local emergency number and migrants often have insufficient knowledge of the health system [60, 64, 67, 75], ii) contacting the local ambulance or calling the emergency number might be challenging for those who do not speak the local language.

Higher utilization of the ED can be ascribed to poor access to PHC services [52, 63, 66]. Host country’s healthcare policies may prevent irregular migrants from accessing PHC services. Other groups of migrants may face barriers when trying to register for PHC services or may be unaware of their entitlement to a GP. The higher rates of admissions and hospitalizations due to ACSC for migrants compared to non-migrants seem to confirm the hypothesis that PHC services are not easily accessible for migrants [56, 60]. Yet, ACSC rates are shown to be high for non-migrants too, suggesting potential structural issues regarding the use of PHC. Additionally, practitioners usually work by appointment and require booking by phone. This is challenging for migrants who do not speak the local language, have strict working schedules, or are employed under irregular contracts that prevent them from requesting time off.

The findings of this systematic review reporting migrants’ lower utilization of the ED compared to non-migrants [51, 55, 57, 69] can be interpreted according to the “healthy immigrant effect”. According to this theory, immigrants have better health outcomes than native-born residents and therefore their need for healthcare, including ED care, would be lower. This is evident in included articles that report lower triage codes among migrants [59, 70], which could mean that migrants are generally in better health conditions compared to natives, regardless of the overall number of ED accesses. Similar results were found in a study conducted in a large urban ED of Parma (Italy) [76] that analyzed ED records from 2008 to 2012 , in which a significantly higher rate of low acuity triage codes was reported for migrants compared to non-migrants. According to the authors, this difference was partially attributable to the younger average age of the migrant population, less affected by the chronic conditions that characterize the local, aging population.

The findings of this review should be interpreted according to the SDH, which have a major impact on people’s health and well-being, and affect migrants’ utilization of healthcare services. In the article by Abdulla et al. [52], immigrant mothers were more likely than non-immigrant ones to visit the ED in the weeks after discharge, as a result of the combined effect of migrant status and poverty. In another included study, unsafe working conditions were the possible cause for the higher rate of ED visits for injuries among refugee men compared to Lebanese men [71]. ED utilization has also been studied specifically in light of patients’ SDH. A study investigating ED use of a Medicaid cohort found that the need for ED care and the number of visits that could have been treated in a PHC setting increased as the SDH characteristics worsened, with patients facing food insecurity, unemployment, and housing instability [77]. Migration is a SDH too, as it significantly influences health outcomes by exposing people to barriers directly related to migratory status, such as fear of deportation and insecure working conditions [78, 79].

Differences in access to public versus private hospitals between migrants and non-migrants [71] can reflect inequalities within highly privatized health systems, where public hospitals provide inpatient acute care and the private sector specializes in more technologically advanced care, which is typically sought by wealthier people. In such cases, access to public services becomes disputed between nationals and refugees, creating tensions, as in the case of Lebanon [80]. The same trend was identified in a multi-country study [81] conducted by the European Social Policy Network (ESPN), which shows that wealthier patients in countries such as Austria, Spain, and Finland often bypass waiting times in the public sector by consulting a practitioner privately and paying out-of-pocket. As a result, waiting times significantly worsen for economically disadvantaged people.

Despite this study not being strictly focused on intragroup differences among migrants, such differences exist, especially concerning documented versus undocumented migrants, and are reported in several studies regarding access to the ED. Ro et al. [82] compared ED utilization between undocumented Latino patients and MediCal-insured Latino patients in Los Angeles, finding a lower rate of ED visits in the former group as compared to the latter (544.25 vs. 571.08). The same study confirmed that undocumented patients experienced a steeper decline in ED utilization during the COVID-19 pandemic than MediCal-insured patients. A 2018 systematic review of studies conducted in Europe [83] reported a lower utilization of healthcare services among undocumented migrants compared to documented migrants. This pattern was often attributed to an existing gap between the health entitlements of undocumented migrants and their service utilization, due to barriers such as lack of awareness, fear, and socioeconomic factors.

To summarize, our systematic review identified several barriers (Supplementary material 3) that could be possible drivers for the inequities experienced by migrants. These can be categorized according to Andersen’s expanded behavioral model of health service use [11].

Among the contextual factors, which can be referred to as “systemic”, there are public charge, fear of discovery [84], safety concerns, low availability of interpreters, long waiting times for a referral, GPs’ working hours, and lack of entitlement to a GP.

When it comes to predisposing characteristics, language was the main hindrance to accessing EDs for migrants across different host countries.

Finally, several enabling factors that can facilitate or impede the utilization of health services - in our case, the ED - were mentioned: low SES, communication issues with providers due to different perceptions of pain and urgency, lack of insurance, lack of knowledge of the local healthcare system, transportation problems, difficulties in obtaining information, lack of family support and loss of previous social networks, as well as precarious working conditions. While the aforementioned barriers are presented as compartmentalized, migrants’ inequities tend to arise from the presence of several barriers reinforcing and influencing each other.

### Recommendations

All the studies included in this review but one adopted a quantitative methodology. More qualitative research engaging both migrants and healthcare providers is needed, as it would allow a deep understanding of migrants’ health-seeking behavior, and their experience when utilizing the ED. We urge authors to present disaggregated data (e.g., age, home country, legal status, SES, and length of stay) in a clear, accurate, and consistent manner to enable the identification of subgroups collectively referred to as “migrants”. To advance research in this field, terms referring to migrants should be used more consistently. Authors tend to rather use terms such as “migrant” and “immigrant” interchangeably, or to apply their own criteria to define this population. While terms such as “asylum seeker” and “refugee” are mostly agreed upon, “migrant” and “immigrant” are typically intended and used at the authors’ discretion.

At the institutional level, we recommend policymakers and health authorities take into consideration inequalities affecting migrants and implement specific interventions to facilitate their access to care. There is a pressing need for tailored and sustainable strategies that consider the diverse health needs of migrants and the deficiencies existing within the healthcare systems of the host countries [85]. Possible strategies include developing health literacy programs, integrating migrants in the development and implementation of health policies, and extending the availability of interpreters and cultural mediators in health facilities [20, 85].

### Strengths and limitations

To the best of our knowledge, this is the first systematic review exploring migrants’ access to the ED without applying geographical restrictions, thus allowing a more comprehensive understanding of the phenomenon. Moreover, by focusing on articles that compare migrant and non-migrant populations, this review provides precious insights into the inequities faced by migrants in host countries. This review also has some limitations. First, no gray literature was included in the search process. Second, the search was restricted only to articles written in English or Italian. Third, the studies included in this review adopted different definitions of migrants, thus preventing a deeper exploration of the factors influencing ED utilization among specific communities. Fourth, the paucity and heterogeneity of included studies prevented their quality appraisal. Nevertheless, details were provided on the type of studies and methodological aspects to enable the reader to understand what studies the results came from. Fifth, the choice of including countries with different health systems and economic conditions may hinder the generalizability of the findings.

## Conclusion

This systematic review gathered and summarized published literature comparing ED utilization between migrant and non-migrant populations to identify differences in access to care and utilization of the ED. Overall, this review highlights that a single pattern of ED utilization by migrants can hardly be identified. There is no consensus on whether migrants access EDs more or less than non-migrants, as well as on whether migrants have more or fewer ED contacts resulting in hospitalization. However, migrants tend to access EDs for less urgent conditions, lack a referral from a GP, access the ED as walk-ins in higher proportions, and are more often discharged AMA, as compared to non-migrants. Higher ED utilization and walk-ins can be attributed to poor access to PHC services. Lower rates of hospitalizations may be associated with migrants’ better health outcomes and lower triage levels, or with difficulties in affording hospitalization-related costs. Language barriers, lack of entitlement to GP services, lack of knowledge of the local healthcare system, as well as other barriers, are significant hindrances to migrants’ effective access to healthcare services.

### Supplementary Information

**Supplementary Material 1.**

**Supplementary Material 2.**

**Supplementary Material 3.**

**Supplementary Material 4.**

**Supplementary Material 5.**

## Data Availability

The datasets used and/or analysed during the current study are available from the corresponding author on reasonable request.

## Abbreviations

ACS: Ambulatory Care Sensitive
ACSC: Ambulatory Care Sensitive Conditions
AMA: Against Medical Advice
ED: Emergency Department
ESB: English-Speaking Non-Native
ESB-BA: English-Speaking Background Born in Australia
ESPN: European Social Policy Network
FW: Foreign Worker
GP: General Practitioner
NESB: Non-English-Speaking Background
NLRC: Non-Latino Legal Residents/Citizens
PHC: Primary Health Care
PRISMA: Preferred Reporting Items for Systematic Reviews and Meta-Analyses
SES: Socioeconomic Status
SDH: Social Determinants of Health
UDLI: Undocumented Latino Immigrants
UNHCR: United Nations High Commissioner for Refugees
US: United States

## References

[1] united-nations-population division. International Migrant Stock | Population Division. https://www.un.org/development/desa/pd/content/international-migrant-stock. Accessed 16 June 2023.

[2] iom. About Migration. https://www.iom.int/about-migration. Accessed 16 June 2023.

[3] UNDRR. Disaster | UNDRR. 2007. https://www.undrr.org/terminology/disaster. Accessed 18 June 2023.

[4] world-health organization. Refugee and migrant health. https://www.who.int/news-room/fact-sheets/detail/refugee-and-migrant-health. Accessed 18 June 2023.

[5] Davies AA, Basten A, Frattini C. Migration: A Social Determinant of the Health of Migrants. International Organization for Migration (IOM). 2009. https://migrationhealthresearch.iom.int/migration-social-determinant-health-migrants. Accessed 20 Mar 2024.

[6] Batalova J. Article: Top Statistics on Global Migration and Mi. | migrationpolicy.org. https://www.migrationpolicy.org/article/top-statistics-global-migration-migrants. Accessed 16 June 2023.

[7] Bongard S, Pogge SF, Arslaner H, Rohrmann S, Hodapp V (2002). Acculturation and cardiovascular reactivity of second-generation Turkish migrants in Germany. J Psychosom Res..

[8] Newbold KB (2006). Chronic Conditions and the Healthy Immigrant Effect: Evidence from Canadian Immigrants. J Ethn Migr Stud..

[9] Gimeno-Feliu LA, Pastor-Sanz M, Poblador-Plou B, Calderón-Larrañaga A, Díaz E, Prados-Torres A (2021). Overuse or underuse? Use of healthcare services among irregular migrants in a north-eastern Spanish region. Int J Equity Health..

[10] Sarría-Santamera A, Hijas-Gómez AI, Carmona R, Gimeno-Feliú LA (2016). A systematic review of the use of health services by immigrants and native populations. Public Health Rev..

[11] Andersen RM (1995). Revisiting the Behavioral Model and Access to Medical Care: Does it Matter?. J Health Soc Behav..

[12] Abubakar I, Aldridge RW, Devakumar D, Orcutt M, Burns R, Barreto ML (2018). The UCL-Lancet Commission on Migration and Health: the health of a world on the move. Lancet..

[13] who. Social determinants of health. https://www.who.int/health-topics/social-determinants-of-health. Accessed 15 Feb 2024.

[14] Galanis P, Spyros K, Siskou O, Konstantakopoulou O, Angelopoulos G, Kaitelidou D. Healthcare services access, use, and barriers among migrants in Europe: a systematic review. medRxiv. 2022. 10.1101/2022.02.24.22271449.

[15] World Health Organization Regional Office for Europe. How health systems can address health inequities linked to migration and ethnicity. World Health Organization. Regional Office for Europe; 2010. https://apps.who.int/iris/handle/10665/345463. Accessed 18 June 2023.

[16] Hacker K, Anies M, Folb BL, Zallman L (2015). Barriers to health care for undocumented immigrants: a literature review. Risk Manag Healthc Policy..

[17] Mona H, Andersson LMC, Hjern A, Ascher H (2021). Barriers to accessing health care among undocumented migrants in Sweden - a principal component analysis. BMC Health Serv Res..

[18] Donnelly TT, Hwang JJ, Este D, Ewashen C, Adair C, Clinton M. If I was going to kill myself, I wouldn’t be calling you. I am asking for help: challenges influencing immigrant and refugee women’s mental health. Issues Ment Health Nurs. 2011;32(5):279–290. 10.3109/01612840.2010.550383. 10.3109/01612840.2010.550383 21574842

[19] Iliadi P (2008). Refugee women in Greece: - a qualitative study of their attitudes and experience in antenatal care. Health Sci J.

[20] World Health Organization Regional Office for Europe. Migration and health: enhancing intercultural competence and diversity sensitivity. World Health Organization. Regional Office for Europe; 2020. https://apps.who.int/iris/handle/10665/332186. Accessed 18 June 2023.

[21] Mangrio E, Sjögren Forss K (2017). Refugees’ experiences of healthcare in the host country: a scoping review. BMC Health Serv Res..

[22] Abood J, Woodward K, Polonsky M, Green J, Tadjoeddin M, Renzaho A (2021). Understanding immigrant settlement services literacy in the context of settlement service utilisation, settlement outcomes and wellbeing among new migrants: A mixed methods systematic review. Wellbeing Space Soc..

[23] Trentin M, Rubini E, Bahattab A, et al. Vulnerability of migrant women during disasters: a scoping review of the literature. Int J Equity Health. 22;135(2023). 10.1186/s12939-023-01951-1. 10.1186/s12939-023-01951-1 PMC10362632 37481546

[24] Da Mosto D, Bodini C, Mammana L, Gherardi G, Quargnolo M, Fantini MP (2021). Health equity during COVID-19: A qualitative study on the consequences of the syndemic on refugees’ and asylum seekers’ health in reception centres in Bologna (Italy). J Migr Health..

[25] Kluge HHP, Jakab Z, Bartovic J, D’Anna V, Severoni S (2020). Refugee and migrant health in the COVID-19 response. Lancet..

[26] World Health Organization. Health emergency and disaster risk management framework. Geneva: World Health Organization; 2019. Section: xi, 31 p. https://apps.who.int/iris/handle/10665/326106. Accessed 18 June 2023.

[27] Credé SH, Such E, Mason S (2018). International migrants’ use of emergency departments in Europe compared with non-migrants’ use: a systematic review. Eur J Public Health..

[28] Gimeno-Feliu LA, Calderón-Larrañaga A, Diaz E, Poblador-Plou B, Macipe-Costa R, Prados-Torres A (2016). Global healthcare use by immigrants in Spain according to morbidity burden, area of origin, and length of stay. BMC Public Health..

[29] Fisher R, Dunn P, Asaria M, Thorlby R. Level or not? - The Health Foundation. https://www.health.org.uk/publications/reports/level-or-not. Accessed 25 Feb 2024.

[30] Boutziona I, Papanikolaou D, Sokolakis I, Mytilekas KV, Apostolidis A (2020). Healthcare Access, Quality, and Satisfaction Among Albanian Immigrants Using the Emergency Department in Northern Greece. J Immigr Minor Health..

[31] Norredam M, Mygind A, Nielsen AS, Bagger J, Krasnik A (2007). Motivation and relevance of emergency room visits among immigrants and patients of Danish origin. Eur J Public Health..

[32] Petersen LA, Burstin HR, O’Neil AC, Orav EJ, Brennan TA (1998). Nonurgent Emergency Department Visits: The Effect of Having a Regular Doctor. Med Care..

[33] De Luca G, Ponzo M, Andrés AR (2013). Health care utilization by immigrants in Italy. Int J Health Care Finance Econ..

[34] Morisod K, Luta X, Marti J, Spycher J, Malebranche M, Bodenmann P (2021). Measuring Health Equity in Emergency Care Using Routinely Collected Data: A Systematic Review. Health Equity..

[35] Trappolini E, Marino C, Agabiti N, Giudici C, Davoli M, Cacciani L (2020). Disparities in emergency department use between Italians and migrants residing in Rome, Italy: the Rome Dynamic Longitudinal Study from 2005 to 2015. BMC Public Health..

[36] Leaman AM, Rysdale E, Webber R (2006). Use of the emergency department by Polish migrant workers. Emerg Med J EMJ..

[37] Lee J, Bruce J, Wang NE (2021). Opportunities for Supporting Latino Immigrants in Emergency and Ambulatory Care Settings. J Community Health..

[38] Russo V, Santarelli S, Magrini L, Moscatelli P, Altomonte F, Cremonesi G (2017). Multicentre Italian analysis on cardiovascular diseases: impact of immigrants’ referral to emergency department. J Cardiovasc Med (Hagerstown, Md)..

[39] Nandi A, Galea S, Lopez G, Nandi V, Strongarone S, Ompad DC (2008). Access to and use of health services among undocumented Mexican immigrants in a US urban area. Am J Public Health..

[40] Watts DJ, Friedman JF, Vivier PM, Tompkins CEA, Alario AJ (2012). Health care utilization of refugee children after resettlement. J Immigr Minor Health..

[41] Müller M, Klingberg K, Srivastava D, Exadaktylos AK (2016). Consultations by Asylum Seekers: Recent Trends in the Emergency Department of a Swiss University Hospital. PLoS ONE..

[42] Deans AK, Boerma CJ, Fordyce J, De Souza M, Palmer DJ, Davis JS (2013). Use of Royal Darwin Hospital emergency department by immigration detainees in 2011. Med J Aust..

[43] Reko A, Bech P, Wohlert C, Noerregaard C, Csillag C (2015). Usage of psychiatric emergency services by asylum seekers: Clinical implications based on a descriptive study in Denmark. Nord J Psychiatry..

[44] Chatzidiakou K, Schoretsanitis G, Schruers KR. Acute Psychiatric Problems among Migrants Living in Switzerland- a Retrospective Study from a Swiss University Emergency Department. Emerg Med Open Access. 2016;6(5). 10.4172/2165-7548.1000338.

[45] Saeki S, Kurosawa Y, Tomiyama K, Tomizawa R, Honda C, Minamitani K. Foreign Patients Visiting the Emergency Department: A Systematic Review of Studies in Japan. JMA J. 2023;6(2):95–103. 10.31662/jmaj.2022-0177. 10.31662/jmaj.2022-0177 PMC10169272 37179726

[46] Lebano A, Hamed S, Bradby H, Gil-Salmerón A, Durá-Ferrandis E, Garcés-Ferrer J (2020). Migrants’ and refugees’ health status and healthcare in Europe: a scoping literature review. BMC Public Health..

[47] Graetz V, Rechel B, Groot W, Norredam M, Pavlova M (2017). Utilization of health care services by migrants in Europe-a systematic literature review. Br Med Bull..

[48] Mahmoud I, Hou XY (2012). Immigrants and the utilization of hospital emergency departments. World J Emerg Med..

[49] Change, Working Group II to the Sixth Assessment Report of the Intergovernmental Panel on Climate. Climate Change 2022: Impacts, Adaptation and Vulnerability. Contribution of Working Group II to the Sixth Assessment Report of the Intergovernmental Panel on Climate Change: Technical Summary. 2022. Cambridge University Press. 10.1017/9781009325844.002.

[50] Page MJ, McKenzie JE, Bossuyt PM, Boutron I, Hoffmann TC, Mulrow CD (2021). The PRISMA 2020 statement: an updated guideline for reporting systematic reviews. BMJ..

[51] Ornelas C, Torres J, Torres J, Alter H, Taira B, Rodriguez R. Anti-immigrant Rhetoric and the Experiences of Latino Immigrants in the Emergency Department. Western J Emerg Med. 2021;22(3). 10.5811/westjem.2021.2.50189. 10.5811/westjem.2021.2.50189 PMC8203025 34125043

[52] Abdulla L, McGowan EC, Tucker RJ, Vohr BR (2020). Disparities in Preterm Infant Emergency Room Utilization and Rehospitalization by Maternal Immigrant Status. J Pediatr..

[53] Huynh MP, Du S, Hanlon C, Yang H, Young A, Ro A (2023). COVID-19-Related Emergency Department Visits Among Undocumented Patients in Los Angeles County. J Health Care Poor Underserved..

[54] Ro A, Huynh MP, Bruckner TA, Du S, Young A (2022). COVID-19 case counts and COVID-19 related Emergency Department visits: differences by immigration status, March-December 2020. BMC Public Health..

[55] Rodriguez RM, Torres JR, Sun J, Alter H, Ornelas C, Cruz M (2019). Declared impact of the US President’s statements and campaign statements on Latino populations’ perceptions of safety and emergency care access. PLoS ONE..

[56] Brandenberger J, Bozorgmehr K, Vogt F, Tylleskär T, Ritz N (2020). Preventable admissions and emergency-department-visits in pediatric asylum-seeking and non-asylum-seeking patients. Int J Equity Health..

[57] Brandenberger J, Pohl C, Vogt F, Tylleskär T, Ritz N (2021). Health care provided to recent asylum-seeking and non-asylum-seeking pediatric patients in 2016 and 2017 at a Swiss tertiary hospital - a retrospective study. BMC Public Health..

[58] Klingberg K, Stoller A, Müller M, Jegerlehner S, Brown AD, Exadaktylos A (2020). Asylum Seekers and Swiss Nationals with Low-Acuity Complaints: Disparities in the Perceived level of Urgency, Health Literacy and Ability to Communicate-A Cross-Sectional Survey at a Tertiary Emergency Department. Int J Environ Res Public Health..

[59] Klukowska-Röetzler J, Eracleous M, Müller M, Srivastava D, Krummrey G, Keidar O (2018). Increased Urgent Care Center Visits by Southeast European Migrants: A Retrospective, Controlled Trial from Switzerland. Int J Environ Res Public Health..

[60] Lichtl C, Lutz T, Szecsenyi J, Bozorgmehr K (2017). Differences in the prevalence of hospitalizations and utilization of emergency outpatient services for ambulatory care sensitive conditions between asylum-seeking children and children of the general population: a cross-sectional medical records study (2015). BMC Health Serv Res..

[61] Sauzet O, David M, Naghavi B, Borde T, Sehouli J, Razum O (2021). Adequate Utilization of Emergency Services in Germany: Is There a Differential by Migration Background?. Front Public Health..

[62] Schwachenwalde S, Sauzet O, Razum O, Sehouli J, David M (2020). The role of acculturation in migrants’ use of gynecologic emergency departments. Int J Gynecol Obstet..

[63] Di Napoli A, Ventura M, Spadea T, Giorgi Rossi P, Bartolini L, Battisti L (2022). Barriers to Accessing Primary Care and Appropriateness of Healthcare Among Immigrants in Italy. Front Public Health..

[64] Di Napoli A, Rossi A, Battisti L, Cacciani L, Caranci N, Cernigliaro A, et al. Valutazione dell’assistenza sanitaria della popolazione immigrata in Italia attraverso alcuni indicatori di un sistema nazionale di monitoraggio. Epidemiol Prev. 2020;44(5-6 Suppl 1):85–93. 10.19191/EP20.5-6.S1.P085.077. 10.19191/EP20.5-6.S1.P085.077 33415950

[65] Henares-Montiel J, Ruiz-Perez I, Mendoza-Garcia O (2018). Health inequalities between male and female immigrants in Spain after the beginning of the economic crisis. Health Soc Care Community..

[66] Rodríguez-Álvarez E, Lanborena N, Borrell LN (2019). Health Services Access Inequalities Between Native and Immigrant in a Southern European Region. Int J Health Serv..

[67] Mahmoud I, Eley R, Hou XY (2015). Subjective reasons why immigrant patients attend the emergency department. BMC Emerg Med..

[68] Etowa J, Sano Y, Hyman I, Dabone C, Mbagwu I, Ghose B (2021). Difficulties accessing health care services during the COVID-19 pandemic inCanada: examining the intersectionality between immigrant status and visible minority status. Int J Equity Health..

[69] Xi S, Song Y, Li X, Li M, Lu Z, Yang Y (2020). Local-Migrant Gaps in Healthcare Utilization Between Older Migrants and Local Residents in China. J Am Geriatr Soc..

[70] Zunino L, Colineaux H, Claudet I, Bréhin C (2021). Description of a migrant pediatric population visiting the Toulouse Children’s Hospital emergency department. Arch Pediatr..

[71] Al-Hajj S, Chahrour MA, Nasrallah AA, Hamed L, Pike I (2021). Physical trauma and injury: A multi-center study comparing local residents and refugees in Lebanon. J Global Health..

[72] Chan JS, Chia DW, Hao Y, Lian SW, Chua MT, Ong ME. Health-seeking behaviour of foreign workers in Singapore:Insights from emergency department visits. Ann Acad Med Singap. 2021;50(4):315–324. 10.47102/annals-acadmedsg.2020484. 33990819

[73] Gulacti U, Lok U, Polat H (2017). Emergency department visits of Syrian refugees and the cost of their healthcare. Pathog Glob Health..

[74] ppic. The Immigration and Citizenship Process. https://www.ppic.org/publication/the-immigration-and-citizenship-process/. Accessed 15 Feb 2024.

[75] Abbott S, Riga M (2007). Delivering services to the Bangladeshi community: the views of healthcare professionals in East London. Public Health..

[76] Zinelli M, Musetti V, Comelli I, Lippi G, Cervellin G. Emergency department utilization rates and modalities among immigrant population. A 5-year survey in a large Italian urban emergency department. Emergency Care J. 2014;10(1). 10.4081/ecj.2014.1896.

[77] McCarthy ML, Zheng Z, Wilder ME, Elmi A, Li Y, Zeger SL (2021). The Influence of Social Determinants of Health on Emergency Departments Visits in a Medicaid Sample. Ann Emerg Med..

[78] Fleischman Y, Willen SS, Davidovitch N, Mor Z (2015). Migration as a social determinant of health for irregular migrants: Israel as case study. Soc Sci Med..

[79] Viruell-Fuentes EA, Miranda PY, Abdulrahim S (2012). More than culture: Structural racism, intersectionality theory, and immigrant health. Soc Sci Med..

[80] Blanchet K, Fouad FM, Pherali T (2016). Syrian refugees in Lebanon: the search for universal health coverage. Confl Health..

[81] Baeten R, Spasova S, Vanhercke B, Coster S. Inequalities in access to healthcare. A study of national policies 2018. 2018. 10.2767/371408.

[82] Ro A, Bruckner TA, Huynh MP, Du S, Young A. Emergency Department Utilization Among Undocumented Latino Patients During the COVID-19 Pandemic. J Racial Ethnic Health Disparities. 2022. 10.1007/s40615-022-01382-8. 10.1007/s40615-022-01382-8 PMC9388205 35982287

[83] Winters M, Rechel B, de Jong L, Pavlova M (2018). A systematic review on the use of healthcare services by undocumented migrants in Europe. BMC Health Serv Res..

[84] Omarzu J (2000). A Disclosure Decision Model: Determining How and When Individuals Will Self-Disclose. Pers Soc Psychol Rev..

[85] Mladovsky P, Rechel B, Ingleby D, McKee M (2012). Responding to diversity: an exploratory study of migrant health policies in Europe. Health Policy (Amsterdam, Netherlands)..

