# Supplementary material for: Exploring differences in the utilization of the emergency department between migrant and non-migrant populations: a systematic review
Source: BMC Public Health. 2024 Apr 5;24:963. doi: 10.1186/s12889-024-18472-3 (PMC10996100; doi:10.1186/s12889-024-18472-3)
Supplement: Supplementary file 1 — Supplementary Material 1. [file 12889_2024_18472_MOESM1_ESM.docx]

**Additional file 1**

**File format**: MS/DOCX

**Title of data**: Tables for Search String

**Description**: The Table shows the search strings used per each database.

| Database | Retrieved records | Search string |
| --- | --- | --- |
| PubMed | **479** | (("transients and migrants"[MeSH Terms] OR "migrant*"[Title/Abstract] OR "immigrant*"[Title/Abstract]) AND ("emergency department"[Title/Abstract] OR "emergency room"[Title/Abstract] OR "emergency service*"[Title/Abstract] OR "emergency care  service*"[Title/Abstract] OR "emergency visit*"[Title/Abstract] OR "emergency outpatient*"[Title/Abstract])) |
| Scopus | **772** | ( TITLE-ABS-KEY ( "migrant*" OR "immigrant*" ) AND TITLE-ABS-KEY ( "emergency department" OR "emergency room" OR "emergency service*" OR "emergency care service*" OR "emergency visit*" OR "emergency outpatient*" ) ) |
| Web of Science | **547** | (TS=("migrant*" OR "immigrant*") AND TS=("emergency department" OR "emergency room" OR "emergency service*" OR "emergency care service*" OR "emergency visit*" OR "emergency outpatient*")) |
