# Supplementary material for: Exploring differences in the utilization of the emergency department between migrant and non-migrant populations: a systematic review
Source: BMC Public Health. 2024 Apr 5;24:963. doi: 10.1186/s12889-024-18472-3 (PMC10996100; doi:10.1186/s12889-024-18472-3)
Supplement: Supplementary file 2 — Supplementary Material 2. [file 12889_2024_18472_MOESM2_ESM.docx]

**Additional file 2**

**File format**: MS/DOCX

**Title of data**: Extraction sheet

**Description**: Extraction sheet used to collect data from retrieved articles.

| General information of the article | Title  Authors  Publication year  Language of the study  Country where the study was conducted |
| --- | --- |
| Study design | Study period  Objective of the study  Study type  Methodology  Study population  Source of data |
| Information about the migrant patients | Legal status  Communities of migrants  Age of migrants  Home country  Host country  Length of stay in host country  Migrants' knowledge of the local language |
| Results of statistical analysis (when no  comparison is made) |  |
| Comparison | Population of comparison  Result of the comparison  Synthesis of the analysis  Vulnerable subgroups |
| Information about the emergency department visit | Health profile/presence of chronic conditions  ED speciality  Triage code  Mode of access  Frequency of use of ED services  Length of stay in the emergency department  Reasons for ED visit  Cost of ED visit |
| Interventions put in place to help migrant patients | Type of intervention  Outcome of intervention |
| Outcome of ED visit | Hospitalization  Ward of destination  Death |
| Migrant patients' perception on the ED stay |  |
| Challenges/barriers | Before ED visit  During ED visit  After ED visit |
| Limitations | Study limitations (authors)  Study limitations (us) |
| Notes |  |
| Articles retrieved from references |  |
