# Supplementary material for: Exploring differences in the utilization of the emergency department between migrant and non-migrant populations: a systematic review
Source: BMC Public Health. 2024 Apr 5;24:963. doi: 10.1186/s12889-024-18472-3 (PMC10996100; doi:10.1186/s12889-024-18472-3)
Supplement: Supplementary file 3 — Supplementary Material 3. [file 12889_2024_18472_MOESM3_ESM.docx]

**Additional File 3.**

**File format**: MS/DOCX

**Title of data**: Table illustrating the barriers reported in the included studies and health system specifications

**Description**: the table contains, for each study included in the review, information regarding the host country, the legal status and home country of the migrants, the barriers reported in the study and a detailed description of the host country’s health system.

| Country | Ref. | Legal status of migrants | Home country | Reported barriers | Health system |
| --- | --- | --- | --- | --- | --- |
| US | Abdulla et al. (2020) | Migrants | Caribbean, Latin America, Asia and Oceania, Africa | Language barriers | The U.S. healthcare system works based on four different patterns: the Beveridge Model, the Bismarck Model, the National Health Insurance Model and the Out of Pocket Model, depending on the population in question. |
|  | Ornelas et al. (2021) | Immigrants | Latin America | Public charge (fear of being denied a green card/visa due to use of certain services) Fear of discovery Communication issues with providers Lack of insurance Safety concerns |  |
|  | Rodriguez et al. (2019) | Immigrants | Latin America | Public charge (fear of being denied a green card/visa due to use of certain services) |  |
| Italy | Di Napoli et al. (2022) | Immigrants | / | Difficulties with GP office hours | Italy’s healthcare system follows the Beveridge model. |
|  | Di Napoli et al. (2020) | Immigrants | / | Language barriers Lack of knowledge of the local system Precarious working conditions |  |
| Switzerland | Klingberg et al. (2020) | Asylum seekers | Eastern Africa, Western Asia, Southern Asia, Northern Africa, Western Africa, Southern Europe. | Language barriers Not having a GP Difficulties with GP office hours Lack of knowledge of the local system | Switzerland’s healthcare system follows the Bismarck Model. |
| Germany | Schwachenwalde et al. (2020) | Migrants | / | Lack of knowledge of the local system | Germany’s healthcare system follows the Bismarck Model. |
|  | Lichtl et al. (2017) | Asylum seekers | / | Lack of knowledge of the local system Language barriers |  |
| China | Xi et al. (2020) | Migrants | / | Lack of insurance  Lack of family support  Low socio-economic status | China’s healthcare system follows, for its urban population, the National Health Insurance Model. For the rural population, it can also follow the Out of Pocket Model. |
| France | Zunino et al. (2021) | Migrants | Western Europe, Eastern Europe, North Africa, Middle East, Africa (outside of North Africa), America | Language barriers Low availability of interpreter services Low socio-economic status Loss of previous social networks | France’s healthcare system is a blend between the Bismarck and Beveridge Models. |
| Canada | Etowa et al. (2021) | Immigrants | / | Long waiting times Difficulty in obtaining a referral Difficulty in obtaining information Transportation problems Language barriers | Canada’s healthcare system follows the National Health Insurance Model. |
| Australia | Mahmoud et al. (2015) | Immigrants | United Kingdom, New Zealand, Republic of Ireland, South Africa, the United States, Canada | Lack of knowledge of the local system | Australia’s healthcare system follows the Beveridge Model. |
