# Supplementary material for: Exploring differences in the utilization of the emergency department between migrant and non-migrant populations: a systematic review
Source: BMC Public Health. 2024 Apr 5;24:963. doi: 10.1186/s12889-024-18472-3 (PMC10996100; doi:10.1186/s12889-024-18472-3)
Supplement: Supplementary file 4 — Supplementary Material 4. [file 12889_2024_18472_MOESM4_ESM.docx]

**Additional file 4**

**File format**: MS/DOCX

**Title of data**: Table illustrating the characteristics of the studies included in the review

**Description**: the table contains, for each study included in the review, information regarding the country, year, study type, sample size, population, objective and results.

| **Ref.** | **Country** | **Year** | **Study type** | **Sample size (migrants)** | **Ref. pop** | **Objective** | **Results** |
| --- | --- | --- | --- | --- | --- | --- | --- |
| Abdulla et al., 2020 | USA | October 2012 - September 2015 | Retrospective cohort study, single-center | 176 immigrant mothers; 203 preterm infants | Non-migrant population | To evaluate the effect of immigrant status on ER visits and rehospitalizations | ER utilization with immigrant status (OR, 1.70; 95% CI, 1.12-2.59); Rehospitalization in immigrant mothers with bronchopulmonary dysplasia and Medicaid (OR, 7.69; 95% CI, 1.80-32.8) are significant strong predictors of rehospitalization. |
| Al-Hajj et al., 2021 | Lebanon | June 2017 - May 2018 | Retrospective descriptive epidemiological study, multicentric | 914 refugees | Non-migrant population | To compare and characterize the ED admissions among local residents and refugees | Injury due to a stab/gunshot (OR) = 3.392, (CI) = 2.605-4.416); concussion injury (OR = 1.718, 95% CI = 1.151-2.565); being injured at work (OR = 4.147, 95% CI = 2.74-6.278); leaving the hospital with injury-related disability (OR=2.271, 95% CI=1.891-2.728)] |
| Brandenberger et al., 2020 | Switzerland | 1st January 2016 - 31st December 2017 | Retrospective cross-sectional study, single-center | 297 ED visits by asylum-seeking children | Other (Non-asylum-seeking pediatric patients) | To compare health visits from asylum-seeking and non-asylum-seeking and the differences in their health needs. | Ambulatory-care-sensitive conditions accounted for 18/149 (12.1%; CI: 0.07–0.18) of the admissions in asylum-seeking and for 1270/11′645 (10.9%; CI: 0.1–0.11) in the non-asylum-seeking patients (p = 0.65; CI: − 0.04-0.06). Non-urgent visits were frequent in asylum-seeking patients with 82% (244/297) of the total visits. |
| Branderberger et al., 2021 | Switzerland | 1st January 2016 - 31st December 2017 | Retrospective cross-sectional study, single-center | 1674 asylum-seeking children | Other (Non-asylum-seeking pediatric patients) | To assess preventable hospital admissions and emergency department visits in asylum-seeking and non-asylum-seeking pediatric patients | ED visits in asylum-seeking patients 19% (317/1674, CI: 0.15–0.23) compared 32% (64,315/200, 642; CI: 0.32–0.32) in the non-asylum-seeking patients (p < 0.001, 95% CI -0.17−-0.09). The proportion of hospital admissions was higher in asylum-seeking patients with 11% (184/1674) compared to 7% (14,692/200,642) in the non-asylum-seeking children (p < 0.00, 95% CI 0.02–0.06). |
| Chan et al., 2021 | Singapore | 1st May 2016 - 31st October 2016. | Retrospective observational study, multicentric | 6.429 FWs | Non-migrant population; Other (non-permanent workers with a residence permit) | To examine the epidemiology, attendance patterns, disposition, and adherence to follow-up, by FWs on work permit | A high proportion of these FWs were triaged to low-acuity status compared to the general ED population (66.9% versus 45.9%, P<0.001). Trauma-related injuries contributed to 34.4% of their visits, and were more likely to result in admission compared to non-trauma-related conditions (18.7% vs 15.2%, P<0.001). |
| Di Napoli et al., 2020 | Italy | 2016-2017 | Retrospective cross-sectional study, multicentric | n/a | Non-migrant population | To evaluate maternal and child care, avoidable hospitalization and access to the emergency room of the immigrant population in Italy. | More often than Italians, immigrant women have during pregnancy: less than 5 gynecological examination (16.3% vs 8.5%), first examination after the 12th week of gestational age (12.5% vs 3.8%), less than 2 ultrasounds (3.8% vs 1.0%). Higher perinatal mortality rates among immigrants compared to Italians (3.6 vs 2.3 x1,000). Higher standardized rates (x1,000) among immigrants compared to Italians of avoidable hospitalization (men: 2.1 vs 1.4; women: 0.9 vs 0.7) and of white triage codes in emergency (men: 62.0 vs 32.7; women: 52.9 vs 31.4). |
| Di Napoli et al., 2022 | Italy | 2016–2017 | Retrospective cross-sectional study, multicentric | 2.410.645 immigrants | Non-migrant population | To evaluate barriers to accessing primary care and the appropriateness of health care among resident immigrants in Italy | Compared to Italian women, immigrant women had fewer than five gynecological examinations (8.5 vs. 16.3%), fewer first examinations after the 12th week of gestational age (3.8 vs. 12.5%), and fewer than two ultrasounds (1.0 vs. 3.8%). Compared to Italians, immigrants had higher standardized rates (× 1,000 residents) of avoidable hospitalizations (males: 2.1 vs. 1.4; females: 0.9 vs. 0.7) and of access to emergency departments for non-urgent conditions (males: 62.0 vs. 32.7; females: 52.9 vs. 31.4). |
| Etowa et al., 2021 | Canada | August 4 - 24, 2020 | Retrospective cross-sectional survey study | 36.674 immigrants/visible minority groups | Other (non-visible minority native-born, visible minority native-born, non-visible minority, immigrants, and visible minority immigrants) | To incorporate both immigrant status and visible minority status for analyzing difficulties accessing health care services during the COVID-19 pandemic | Main results show that, compared to white native-born, visible minority immigrants are less likely to report difficulties accessing non-emergency surgical care (OR=0.55, p <0.001), non-emergency diagnostic test (OR=0.74, p <0.01), dental care (OR=0.71, p <0.001), mental health care (OR=0.77, p <0.05), and making an appointment for rehabilitative care (OR=0.56, p <0.001) but more likely to report difficulties accessing emergency services/urgent care (OR=1.46, p <0.05). |
| Gulacti et al., 2017 | Turkey | January 1st - December 31st 2015 | Retrospective observational study, single-center | 9842 refugees | Other (general population accessing the ED in 2010) | To evaluate the demographic and clinical characteristics of ED visits made by Syrian refugees and to assess the cost of their healthcare | The number of ED visits significantly increased in 2015 compared with 2010; the increase in the proportion of total ED visits was 8% (n = 11,275, dif: 8%, CI 95%: 7.9– 8.2, p < 0.001). Of this 8%, 6.5% were visits made by Syria refugees and the remaining 1.5% accounted for the visits made by other individuals. Upper respiratory tract infections (URTI) were the diseases most frequently presented (n = 4,656; 47.3%), and 68.5% of ED visits were inappropriate (n = 6,749). The median ED length of stay (LOS) of the Syrian refugees was significantly longer than that of the other individuals visiting the ED (p < 0.001). The total cost of the healthcare of the Syrian refugees who visited the ED was calculated as US$ 773,374.63. |
| Henares-Montiel et al., 2018 | Spain | 2014 | Retrospective cross‐sectional study | n/a | Non-migrant population | To analyze health inequalities in the immigrant population in Spain in 2014, while differentiating between immigrants and natives | The result of the comparison between immigrants and natives is not statistically significant for the use of emergency services. |
| Huyn et al., 2023 | USA | March 20, 2020 - September 30, 2020 | Retrospective cross-sectional study, single-center | 10.203 undocumented migrant people | Other (patients with a Medi-Cal plan as their primary insurance type) | To compare the probability of COVID-19-related ED visits between undocumented immigrants and Medi-Cal patients, and to examine differences in these comparisons over time. | Undocumented patients had higher odds of COVID-19-related ED visits than Medi-Cal patients (OR: 1.41, 95% CI: 1.24–1.60) for all months in the study period except September. |
| Klingberg et al., 2020 | Switzerland | December, 2016– July, 2017 | Prospective cross-sectional, controlled, single-center | 106 Asylum-seeking people | Non-migrant population | To conduct interviews with AS(Asylum seeker) and SN(Swiss nationals) patients, in order to understand the different factors that influence consultations in the ED for non-urgent conditions | AS and SN differed in their reasons for seeking care in the ED, their knowledge of the Swiss HCS, and their perceptions of medical urgency. However, other factors, such as length of stay, discharge type, and time of visit did not differ between the two groups. AS patients’ has lower levels of knowledge of the Swiss HCS, high usage of the ED and primary care services by AS patients. Even though the majority of both groups sought care in the ED without prior consultation by a GP, the perceived level of urgency among SN was closer to the assessment of the attending physicians. The level of education reported by the AS group was significantly lower than in the SN group. Furthermore, the work status differed significantly, as most AS patients were unemployed, but most SN were employed or self-employed. Low socioeconomic status is linked to the overuse of ED care. |
| Klukovska-Röetzler et al., 2018 | Switzerland | January, 2013 - December, 2017 | Retrospective cohort study, single-center | 12.852 immigrant people | Non-migrant population | To describe the characteristics of SE immigrant patients admitted to our ED, in comparison to Swiss patients; To compare types of referral, reasons for admission, and triage of Swiss and SE patients | There was a significant association between the triage level and immigration from SE (p < 0.0001). The mean triage level in patients from SE was 2.84 (95% CI: 2.82–2.85), but from Switzerland 2.61 (95% CI: 2.60–2.61) (p < 0.001). Following the ED consultation, 65.5% of Swiss patients were treated as outpatients and 34.5% were hospitalized. SE immigrant patients were hospitalized less often (21.0%). Self-referral group, which was more frequent with the SE immigrants (59.9%) than in the Swiss patient population (41.2%), In contrast, referral by ambulance was more frequent in the Swiss patients than in the SE group (16.2% vs. 7.7%). Swiss patients were transferred twice more often from an external hospital or an external doctor than SE patients (external hospital, Swiss 7.0% vs. SE 3.6%; external doctor Swiss 7.0% vs. SE 3.0%) A highly significant association was found between ‘reason for admission’ and immigration from SE (p < 0.001). About 55.0% of Swiss patients (55.3%, n = 84,717) presented with internal medical complaints, 29.5% (n = 45,284) with surgical complaints, and 4.5% with psychiatric complaints. Almost 10.0% of Swiss patients (9.9%, n = 15,127) used a medical service in the fast track section of ED. These values differed significantly in the SE immigrant group: medical: 48.2% (n = 6326), surgery: 26.4% (n = 3388), psychiatry: 4.4% (n = 559), and fast track: 18.9% (n = 2423) . |
| Lichtl et al., 2017 | Germany | 2015 | Retrospective cross-sectional study, single-center | 644 Asylum-seekers | Non-migrant population | To assess the differences between asylum-seeking children and children of the general population | The odds of utilization of emergency outpatient services for ACS conditions were 4.89 times [95% CI: 4.1; 5.85] higher among asylum-seeking children than among children of the general population. As for hospitalizations, 1 to 3 year olds were the most likely age group to make use of emergency services for ACS conditions (OR = 1.19 [95% CI: 1.0; 1.42]). In emergency outpatient care, the odds of service utilization for ACS conditions by the asylum-seeking population were 4.93 times [4.11; 5.91] the odds of the general population. |
| Mahmoud et al., 2015 | Australia | August 2012 - December 2012. | Prospective cross-sectional study | 231 patients from a non-English speaking background (NESB) | Other (ESB-BA: English speaking background born in Australia; ESB-NBA: English speaking background not born in Australia; NESB: Non English-speaking background) | To investigate the subjective reasons why different groups of immigrants attended the ED | Compared to ESB-BA patients NESB patients were less likely to consider contacting a general practitioner (GP) before attending the ED (Odds Ratios (OR) 0.6 (95% Confidence Interval (CI) 0.4–0.8, p < .05) While ESB-NBA were more likely to consider contacting a GP 1.7 (1.1–2.5, p < .05). Both the NESB patients and the ESB-NBA patients were far more likely than ESB-BA patients to report that they had visited the ED either because they do not have a GP (OR 7.9, 95% CI 4.7–13.4, p < .001) and 2.2 (95% CI 1.1–4.4, p < .05) respectively and less likely to think that the ED could deal with their problem better than a GP (OR 0.5 (95% CI 0.3–0.8, p < .05) and 0.7 (0.3–0.9, p < .05) respectively. The NESB patients also thought it would take too long to make an appointment to consult a GP (OR 6.2, 95% CI 3.7–10.4, p < 0.001). |
| Ornelas et al., 2021 | USA | mid-June 2017 - mid-December 2018 | Grounded theory study, multicentric | 129 Undocumented Latino Immigrants (UDLI) | Other (UDLI: undocumented Latino immigrants; LLRC: legal Latino residents/citizens; NLRC: non-Latino legal residents) | To provide further nuance and details regarding the experience of undocumented patients in the ED, including the fear of accessing emergency care, by surveying patients during real-time ED care. | Fear related to immigration status can serve as a barrier to ED care for patients, especially for undocumented immigrants.Beyond fear of discovery, UDLI mentioned other barriers to care, mainly healthcare expenses. |
| Ro et al., 2022 | USA | March - December 2020 | Retrospective cross-sectional study, multicentric | 19.615 undocumented patients | Other (Medi-Cal insured, legal immigrant/US born) | To examine undocumented immigrants’ potential ED underutilization relative to documented patients | Undocumented patients were also more likely to have a COVID-19 related ED visit than Medi-Cal patients during the study period (OR=1.37, 95% CI=1.24–1.52). The Medi-Cal patients were less sensitive to rising COVID-19 cases than undocumented immigrant patients. For undocumented patients, the probability of a COVID-19 related ED visit increased as case count increased. Conversely, the predicted probabilities of a COVID-19 related visit remained relatively flat over increasing COVID-19 cases for Medi-Cal patients. |
| Rodriguez et al., 2019 | USA | mid-June 2017 - mid-December 2018 | Prospective cross-sectional study | 452 Undocumented Latino Immigrant (UDLI) | Other (UDLI: undocumented Latino immigrants; LLRC: legal Latino residents/citizens; NLRC: non-Latino legal residents) | To determine whether the statements about deportation and denying services to undocumented immigrants made them afraid to access the ED for care | Of the UDLI who reported experiencing fear, 55% stated that they delayed coming to the ED (median delay of 2–3 days). Similar percentages of UDLI (24%, 95% CI 21–28%) and LLRC (26%, 95% CI 22–30%) reported that they had friends or family members who had not come to the ED because of fear of discovery, greater than the 15% (95% CI 11–19%) of NLRC who reported having friends/family who had not come to the ED. Only undocumented status (aOR 2.8; 95% CI 2.1–3.9), belief that the president’s immigrant statements are being/will be enacted (aOR 2.4; 95% CI 1.6–3.5), and identification as Latino (aOR 1.5; 95% CI 1.1–2.1) were independently associated with feeling unsafe in the US. Only undocumented status (aOR 7.4; 95% CI 4.4–13.3) and belief that the president’s immigrant statements are being/will be enacted (aOR 2.1; 95% CI 1.0 to 4.8) were independently associated with fear of coming to the ED |
| Rodriguez-Alvarez et al., 2019 | Spain | 2013 - 2014 | Retrospective cross-sectional study | 1.908 immigrant people | Non-migrant population | To examine the inequalities in access to different levels of health care services according to place of birth | There is a higher probability of using general practitioner services in immigrant women (PR: 1.19; 95% CI: 1.12–1.26) and men (PR: 1.11; 95% CI: 1.01–1.23) than in natives. |
| Sauzet et al., 2021 | Germany | July 2017 - July 2018 | Retrospective cross-sectional study, multi-centric | 633 1st generation migrants | Other (patients with different migration status) | To assess the utilization of emergency services among 1st and 2nd generations migrant and natives. | 1st generation migrant patients (n = 633) had significantly lower odds than non-migrants to have an adequate utilization of services [OR 0.78, 95% confidence interval (0.62, 0.99), p-value 0.046]. For 2nd generation patients (n = 268), no statistically significant difference was found [OR 0.80, 95% confidence interval (0.56, 1.15), p-value 0.231]. |
| Schwachenwalde et al., 2020 | Germany | 2017 - 2018 | Prospective cross-sectional study | 477 migrant people | Non-migrant population | To examine whether acculturation of migrant patients is a predictor of non-urgent use of gynecologic emergency departments (GEDs). | Low acculturation was a significant predictor of non-urgent use if defined only by health system criteria (adjusted odds ratio [AOR], 1.58; 95% confidence interval [CI], 1.02–2.44; P=0.041). Inversely, low acculturation had a significant negative effect on non-urgent use if defined only by patient criteria (AOR, 0.58; 95% CI, 0.38–0.90; P=0.014). |
| Xi et al., 2020 | China | 2016 | Prospective cross-sectional study | 1920 older migrants | Non-migrant population | To quantify the disparities in healthcare utilization between older migrants and local residents. | Older migrants were less likely to utilize outpatient (odds ratio [OR] = 0.757; 95% confidence interval [CI] = 0.617-0.928), inpatient (OR = 0.642; 95% CI = 0.443-0.931), and preventive care (OR = 0.743; 95% CI = 0.643-0.858) and were more likely to use medication (OR = 1.254; 95% CI = 1.089-1.445) than local residents. Differences in ER admissions and dental care utilization were not significant in the regression analysis. |
| Zunino et al., 2021 | France | 2018 | Retrospective observational study, single-center | 203 migrant children | Non-migrant population | To describe the sociodemographic and medical characteristics of a pediatric migrant population visiting an ED | The severity of the reasons for visiting (90% of the reasons for visiting had a CCMU (Clinical Classification of Emergency Patients) of 1 or 2) and the hospitalization rate (9%) were not higher in the pediatric migrant population than in the general pediatric population (actually lower). There was a language barrier in 78% of the visits analyzed with underuse of professional interpreting (7%). |
