# Supplementary material for: Exploring differences in the utilization of the emergency department between migrant and non-migrant populations: a systematic review
Source: BMC Public Health. 2024 Apr 5;24:963. doi: 10.1186/s12889-024-18472-3 (PMC10996100; doi:10.1186/s12889-024-18472-3)
Supplement: Supplementary file 5 — Supplementary Material 5. [file 12889_2024_18472_MOESM5_ESM.docx]

**Additional file 5**

**File format**: MS/DOCX

**Title of data**: Table illustrating the demographic characteristics of the samples

**Description**: the table contains, for each study included in the review, information regarding the sample population, namely legal status, mean age, years, home country, host country, length of stay in the host country, knowledge of the local language.

| Reference | Legal status | Mean age, years (dispersion) | Home country | Host Country | Length of stay in the host country (% of the sample) | Knowledge of the local language |
| --- | --- | --- | --- | --- | --- | --- |
| Abdulla et al., 2020 | Migrant | 30,6 (± 6) | Africa Asia and Oceania Caribbean Latin America | USA | <5 years: 22% 5-9 years: 26% 10-14 years: 19% ≥ 15 years: 33% | The language of the host country was not the primary one in 60% of the sample |
| Al-Hajj et al., 2021 | Refugee | 35.1 (±23.9) | Syria | Lebanon | n/a | n/a |
| Brandenberger et al., 2020 | Asylum-seeker | n/a | Afghanistan  Eritrea Syria | Switzerland | n/a | n/a |
| Branderberger et al., 2021 | Asylum-seeker | n/a | Afghanistan  Eritrea Iraq Syria  Somalia | Switzerland | n/a | n/a |
| Chan et al., 2021 | Foreign worker | Voluntary admission migrants: 36.78 (SD 10.61) Compulsory admission migrants: 36.78 (SD 10.61) | Bangladesh Malaysia China India the Philippines Indonesia Myanmar | Singapore | n/a | n/a |
| Di Napoli et al., 2020 | Immigrant | 30,3 | n/a | Italy | n/a | n/a |
| Di Napoli et al., 2022 | Immigrant | n/a | n/a | Italy | n/a | n/a |
| Etowa et al., 2021 | Immigrant | n/a | n/a | Canada | n/a | n/a |
| Gulacti et al., 2017 | Refugee | 34.6 (± 15.2 ) | Syria | Turkey | n/a | n/a |
| Henares-Montiel et al., 2018 | Immigrant | Male: 41.40 (SD 13.97); Female: 41.49 (SD 14.35) | n/a |  | n/a | n/a |
| Huyn et al., 2023 | Undocumented | 25 (21–37) | Hispanic/Latino | USA | n/a | n/a |
| Klingberg et al., 2020 | Asylum-seeker | 39.70 (SD= 15.87) | Eastern Africa Western Asia Southern Asia Northern Africa Western Africa | Switzerland | n/a | No knowledge of the languages spoken in the host country: 53.8% of the sample |
| Klukovska-Röetzler et al., 2018 | Immigrant | 39.70 (SD = 15.87) | Albania Bosnia and Herzegovina Bulgaria Croatia Greece Hungary Kosovo Macedonia Moldova Montenegro Romania Serbia Slovenia Turkey | Switzerland. | n/a | n/a |
| Lichtl et al., 2017 | Asylum-seeker | n/a | n/a | n/a | Average length of stay in 2016: ≥ 4 weeks | n/a |
| Mahmoud et al., 2015 | Immigrant | n/a | United Kingdom New Zealand Republic of Ireland South Africa United States Canada | Australia | n/a | Migrants speaking the local language (English): n=597; migrants not speaking the local language: n=231 * |
| Ornelas et al., 2021 | Immigrant | n/a | Latin America | USA | n/a | Local language (English) as migrants’ primary language: 32%; Language of the sending country (Spanish) as migrants’ primary language: 68% |
| Ro, Huyn et al., 2022 | Undocumented | n/a | Hispanic | USA | n/a | n/a |
| Rodriguez RM et al., 2019 | Immigrant | n/a | Latin America | USA | n/a | Migrants’ reporting little to no proficiency of the local language (English): 78% |
| Rodriguez-Alvarez et al., 2019 | Immigrant | (range: 16-59) | Latina America  Basque Country | Spain | < 5 years: 13.9% 5-10 years: 49.4%  >10 years: 36.7% | 91.4% was fluent/had no difficulty understanding the local language; 8.96% had difficulty understanding the local language/understood very little |
| Sauzet et al., 2021 | Migrant | 1st generation: 42 years (SD: 16), 2nd generation 32 (SD:12) | European Union Turkey Russia non-EU eastern Europe Middle East North and South America Asia and Africa | Germany. | n/a | n/a |
|  |  |  |  |  |  |  |
| Schwachenwalde et al., 2020 | Migrant | 33.5 (SD available by each category) * | n/a | Germany | n/a | Percentage of migrants not speaking the local language (German): 84.9% * |
| Xi et al., 2020 | Migrant | n/a | n/a | China | n/a | n/a |
| Zunino et al., 2021 | Migrant | n/a | Most frequent:  Albania Bulgaria Syrian  Regions: Western Europe Eastern Europe North Africa Middle East Africa America | France | Less than 1 month: 15.9% 1 month to 1 year: 47.9%  More than 1 year: 9.2% | n/a |

*Number calculated by the authors
